# BMP8B Activates Both SMAD2/3 and NF-κB Signals to Inhibit the Differentiation of 3T3-L1 Preadipocytes into Mature Adipocytes

**DOI:** 10.3390/nu16010064

**Published:** 2023-12-25

**Authors:** Shenjie Zhong, Xueqing Du, Jing Gao, Guangdong Ji, Zhenhui Liu

**Affiliations:** 1 College of Marine Life Sciences, Institute of Evolution & Marine Biodiversity, Ocean University of China, Qingdao 266003, China; zsj2879590908@163.com (S.Z.); duxueqing0316@163.com (X.D.); gj15069890865@163.com (J.G.); jamesdong@ouc.edu.cn (G.J.); 2 Laoshan Laboratory, Qingdao 266237, China

**Keywords:** BMP8B, 3T3-L1, adipocyte differentiation, SMAD2/3, NF-κB

## Abstract

Bone morphogenetic protein 8B (BMP8B) has been found to regulate the thermogenesis of brown adipose tissue (BAT) and the browning process of white adipose tissue (WAT). However, there is no available information regarding the role of BMP8B in the process of adipocyte differentiation. Here, we showed that BMP8B down-regulates transcriptional regulators PPARγ and C/EBPα, thereby impeding the differentiation of 3T3-L1 preadipocytes into fully mature adipocytes. BMP8B increased the phosphorylation levels of SMAD2/3, and TP0427736 HCl (SMAD2/3 inhibitor) significantly reduced the ability of BMP8B to inhibit adipocyte differentiation, suggesting that BMP8B repressed adipocyte differentiation through the SMAD2/3 pathway. Moreover, the knockdown of BMP I receptor ALK4 significantly reduced the inhibitory effect of BMP8B on adipogenesis, indicating that BMP8B triggers SMAD2/3 signaling to suppress adipogenesis via ALK4. In addition, BMP8B activated the NF-κB signal, which has been demonstrated to impede PPARγ expression. Collectively, our data demonstrated that BMP8B activates both SMAD2/3 and NF-κB signals to inhibit adipocyte differentiation. We provide previously unidentified insight into BMP8B-mediated adipogenesis.

## 1. Introduction

Excessive food intake and/or reduced energy expenditure may facilitate the proliferation of adipocytes, resulting in obesity. Adipocytes originate from multipotent mesenchymal stem cells (MSCs). MSCs first transform into preadipocytes and subsequently differentiate into mature adipocytes. Three distinct stages can be divided for the process of adipogenesis: (1) commitment of MSCs to the lineage of adipocytes; (2) clonal expansion involving DNA and cell replication; (3) terminal differentiation, which involves the activation of specific transcription factors such as the peroxisome proliferator-activated receptor gamma (PPARγ) and the CCAAT/enhancer-binding protein (C/EBP) family. Along with this terminal differentiation stage, there is a significant rise in the expression of genes involved in adipogenesis, such as adipocyte fatty acid binding protein (FABP), fatty acid synthase (FAS), and acetyl CoA carboxylase (ACC) [1,2,3,4]. The process of adipogenesis is also affected by other factors, of which bone morphogenetic proteins (BMPs) have recently emerged as significant contributors [5,6,7,8].

The transforming growth factor-β (TGF-β) superfamily, to which BMPs belong, is highly conserved across vertebrates [9,10]. Initially, they were discovered for their ability to induce cartilage and bone formation [11]. BMPs now have recognized roles in adipose tissue development and adipogenic differentiation [12,13,14,15,16]. BMPs play different roles in adipogenesis, which are influenced by various factors, such as cellular stages, BMP dosages, and BMP types [17]. BMP2, BMP4, and BMP6 can cause pluripotent stem cells to specialize into adipogenic lineages [18,19,20], but 3T3-F442A preadipocytes treated with BMP2 showed insulin-induced reductions in lipid accumulation [21]. Overexpression of BMP4 reduces the size and mass of white adipose tissue (WAT) and stimulates the browning of WAT. Additionally, BMP4, BMP7, and BMP9 have been found to enhance the formation of brown adipose tissue (BAT), increase thermogenesis in BAT, and aid in the development of beige adipocytes [22,23,24,25]. On the other hand, BMP3B has been found to inhibit the formation of adipocytes in 3T3-L1 cells [26,27].

It has been observed that in *Mus musculus*, two *Bmp8* genes are present, namely *Bmp8a* and *Bmp8b* [28]. In zebrafish, BMP8A has been shown to be important in lipid metabolism [29]. Very recently, BMP8A has shown the ability to inhibit adipogenesis [30]. As for BMP8B, it may modulate energy metabolism by enhancing BAT thermogenesis by activating the AMPK pathway [31,32,33]. Reduced metabolic rates and impaired thermogenesis have been observed in *Bmp8b^−/−^* mice [33]. Despite the high expression of BMP8B in BAT, its expression in WAT is relatively low, so little attention has been paid to the function of BMP8B in white adipocyte differentiation. In this work, we explore the potential capacity of BMP8B in adipocyte differentiation. For the first time, we demonstrated that BMP8B inhibits adipogenesis via the SMAD2/3 and NF-κB pathways. Additionally, BMP8B increases TNF signaling and NOD-like receptor signaling, both of which contribute to immune regulation. Our data provide additional evidences for the link between the regulation of the immune system and differentiation of adipocytes.

## 2. Materials and Methods

### 2.1. Antibodies

Affinity Biosciences: p-SMAD1/5/8 at Ser463 + Ser465 (AF8313; 1:1000), SMAD1/5/8 (AF0614; 1:1000); p-SMAD2/3 at Thr8 (AF3367; 1:1000); SMAD2/3 (AF6367; 1:1000); C/EBPα (AF6333; 1:1000); p-ERK1/2 at Thr202/Tyr204 (AF1015; 1:1000); ERK1/2 (AF0155; 1:1000); p-p65 at Ser536 (AF2006; 1:1000); p65 (AF5006; 1:1000); p-IKKα/β at Ser180/Ser181 (AF3013; 1:1000); IKKα/β (AF6014; 1:1000). Sangon Biotech: p-ACC at Ser79 (D155180; 1:1000); ACC (D155300; 1:1000). Abcam: BMP8B (ab181253; 1:1000). CWBIO: Goat Anti-Rabbit IgG, HRP Conjugated (CW0103S; 1:4000). Bioss: PPARγ (bs-4590R; 1:1000); p-p38 MAPK at Thr180 + Tyr182 (bs-2210R; 1:1000); p38 MAPK (bs-0637R; 1:1000); β-actin (bs-0061R; 1:2000); p-JNK at Thr183 + Tyr185 (bs-1640R; 1:1000); JNK (bs-2592R; 1:1000).

### 2.2. Cell Culture and Differentiation

3T3-L1 and HEK293T cells were provided by ATCC company. The cells were cultured in DMEM supplemented with 10% (vol/vol) FBS and 1% (vol/vol) penicillin/streptomycin.

48 h after reaching 100% confluency (defined as Day 0), cells were treated with MDI cocktail (1 μg/mL insulin, 0.25 μM DEX, and 0.5 mM IBMX) for another two days. Subsequently, cells were treated with insulin (1 μg/mL final concentration) again for 48 h. Moving forward, the medium was refreshed every two days.

### 2.3. qRT-PCR

qRT-PCR was executed as previously described [30]. In general, TRIzol reagent (Life Technologies, New York, NY, USA) was employed to extract total RNA. The cDNA was prepared using the Hifair^®^ AdvanceFast One-step RT-gDNA Digestion SuperMix for qPCR. qRT-PCR was performed on an ABI 7500 Fast Real-Time PCR System using ChamQ SYBR Color qPCR Master Mix. The *Gapdh* was used to normalise mRNA levels. The primers for qRT-PCR can found in Table 1.

### 2.4. Lentiviral

To generate lentivirus, HEK293T cells were transfected with pLVX-shRNA2 or pLVX-mCMV-ZsGreen1-Puro, along with psPAX2 and pMD2.G. Subsequently, 3T3-L1 cells were infected with lentivirus for 48 h. To identify stable cells, the culture medium was supplemented with 2 μg/mL puromycin and renewed every 48 h. The surviving cells were amplified and confirmed through Western blotting.

### 2.5. Luciferase Activity Assay

In general, HEK293T cells were co-transfected with plasmids (luciferase reporter/pRL-TK/expression plasmid) for two days. Subsequently, the Dual Luciferase Reporter Gene Assay Kit (Yeasen, Shanghai, China) was employed to quantify the luciferase activity. To ensure comparability, the obtained data were normalized through the computation of the Firefly/*Renilla* luciferase activity ratio.

### 2.6. RNA Sequencing

Three independent samples were collected from each group, namely, Group 1 (LV-ZsGreen1) and Group 2 (LV-*Bmp8b*), for the purpose of RNA sequencing analysis. The sequencing was carried out by Biomarker Technologies Corporation. KEGG pathway enrichment analyses were performed on MKCloud (www.biocloud.net, accessed on 22 November 2021).

### 2.7. Western Blot

Cells were lysed using NP-40 buffer (protease inhibitor cocktail). The lysates were then subjected to SDS-PAGE and transferred onto PVDF membranes, immunoblotted with specific antibodies, and detected with the Omni-ECL™Femto Light Chemiluminescence Kit. Visualization of the membrane was achieved with a fluorescent Western blot imaging system. Equal loading of the proteins was confirmed through the detection of β-actin. For quantification of the immunoblot, ImageJ software (V 1.8.0) was used.

### 2.8. Plasmid Construction

Some plasmids including pCMV-*Alk2*, pCMV-*Alk3*, pCMV-*Alk4*, pCMV-*Alk5*, pCMV-*Alk7*, pCMV-*Acvr2a*, pCMV-*Acvr2b*, pCMV-*Bmpr2*, and pCMV-*Tgfβr2* were described previously [30]. The ORF of mouse *Bmp8b* was cloned into the pCMV-C-Flag or pLVX-mCMV-ZsGreen1-Puro vector. The sequences shRNA-*Bmp8b#1*: GCTCTACTATGATAGAAACAATTCAAGAGATTGTTTCTATCATAGTAGAGCTTTTTT; shRNA-*Bmp8b#2*: GCCTTTCATGGTTGGTTTCTTTTCAAGAGAAAGAAACCAACCATGAAAGGCTTTTTT; and shRNA-*Bmp8b#3*: GCTGACCTGATTATGAGCTTTTTCAAGAGAAAAGCTCATAATCAGGTCAGCTTTTTT were cloned to pLVX-shRNA2-Puro vector, respectively. Dominant negative mutant plasmids, including pLVX-mCMV-ZsGreen1-*Alk3*-△GS, pLVX-mCMV-ZsGreen1-*Alk4*-△GS, and pLVX-mCMV-ZsGreen1-*Alk5*-△GS, were described previously [30]. All constructs were validated through DNA sequencing. The primers are listed in Table 2.

### 2.9. Oil Red O Staining

To begin the experiment, the cells underwent two rounds of PBS washing, followed by a 20 min fixation with 4% PFA. Subsequently, the fixed cells were subjected to 1 min of washing with 60% isopropanol and stained with Oil Red O. The cells were washed with 60% isopropanol and rinsed two times with distilled water. Finally, cells were observed under a microscope and photographed. To quantify the Oil Red O, the dye was extracted using isopropanol, and an absorbance reading was conducted at the wavelength of OD_492_ nm.

### 2.10. Chemical Inhibition

DMH1 (Selleck), TP0427736 HCl (Selleck), and JSH-23 (Selleck) inhibitors were dissolved in DMSO, respectively. These were diluted directly into the medium at a final concentration of 5 μM.

### 2.11. Statistics and Analysis

The experiments were repeated at least three times. Statistical analysis was conducted using GraphPad Prism version 8.0.2 software. The statistical data were shown as the mean ± SD. One-way ANOVA was executed to analyse the *p* value. ns, not significant; * *p* < 0.05; ** *p* < 0.01; *** *p* < 0.001. 

## 3. Results

### 3.1. BMP8B Suppresses Adipocyte Differentiation

**Figure 1:**
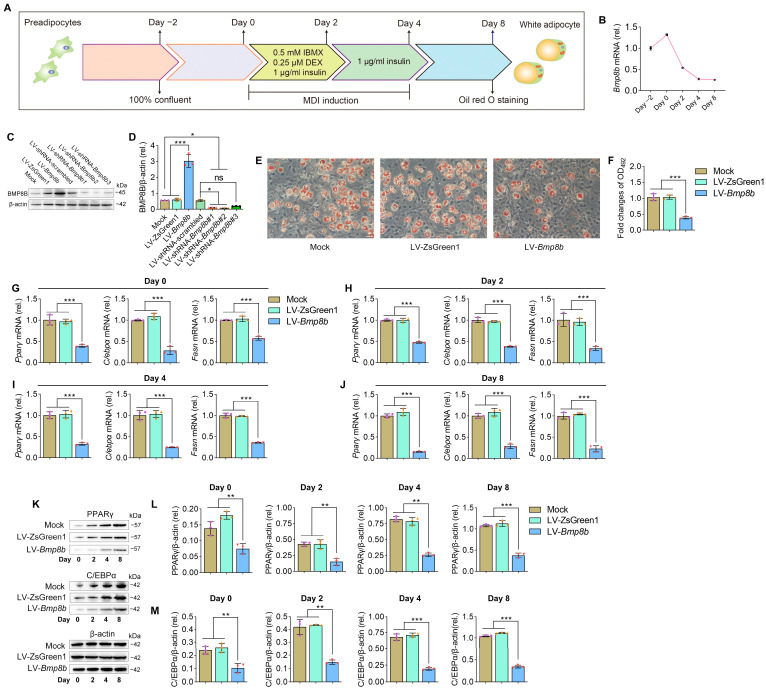
Overexpressing BMP8B inhibits adipocyte differentiation. (**A**) 3T3-L1 preadipocyte differentiation protocol. (**B**) The expression pattern of *Bmp8b* during adipogenesis. (**C**,**D**) Effective increased and inhibited *Bmp8b* expression in 3T3-L1 adipocytes. (**E**) On Day 8, Oil Red O staining revealed lipid contents in cells (Mock, LV-ZsGreen1, and LV-*Bmp8b*). (**F**). Quantification of lipid content in cells after adipogenic differentiation (Day 8). (**G**–**J**) qRT-PCR analyses of the expression of adipogenic marker genes (*Cebpα*, *Pparγ*, and *Fasn*) on Day 0 (**G**), Day 2 (**H**), Day 4 (**I**), and Day 8 (**J**). (**K**–**M**) Protein levels of PPARγ (**L**) and C/EBPα (**M**) detected through immunoblotting. β-actin was used as a loading control. ImageJ software was used to quantify protein levels. Scale bar = 20 µm. The symbols in the charts represent three biological replicates. The data were presented as mean ± SD and analyzed using one-way ANOVA (ns not significant, * *p* < 0.05, ** *p* < 0.01, *** *p* < 0.001).

**Figure 2:**
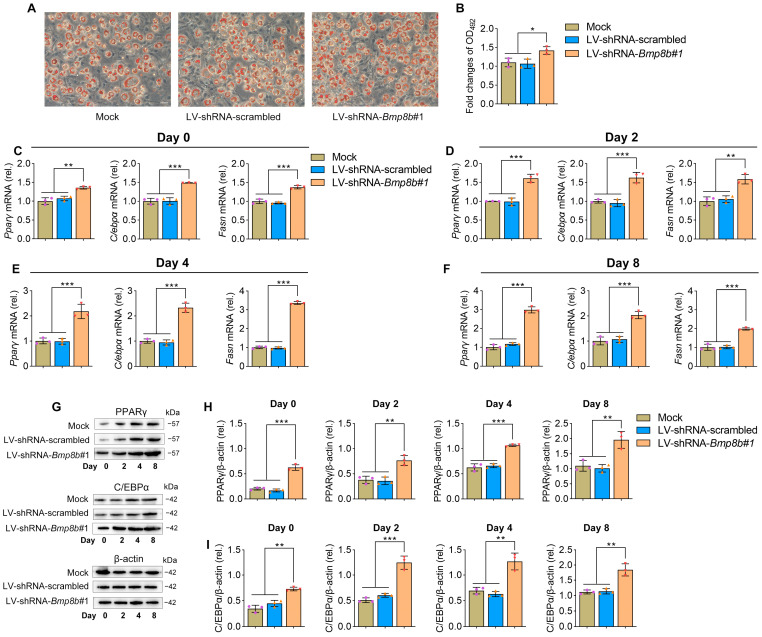
Knockdown BMP8B promotes adipogenesis. (**A**) On Day 8, Oil Red O staining revealed lipid contents (Mock, LV-shRNA-scrambled, and LV-shRNA-*Bmp8b#1*). (**B**) Quantification of lipid content in cells after adipogenic differentiation (Day 8). (**C**–**F**) qRT-PCR analyses of the expression of adipogenic marker genes (*Cebpα*, *Pparγ*, and *Fasn*) on Day 0 (**C**), Day 2 (**D**), Day 4 (**E**), and Day 8 (**F**). (**G**–**I**) On the day after induction, the protein levels of PPARγ (**H**) and C/EBPα (**I**) were detected through immunoblotting. Scale bar = 20 µm. The symbols in the charts represent three biological replicates. The data were presented as mean ± SD and analyzed using one-way ANOVA (* *p* < 0.05, ** *p* < 0.01, *** *p* < 0.001).

No information is currently available regarding the involvement of BMP8B in the process of adipogenesis. By treating 3T3-L1 cells with specific hormones, they can be induced to transform into adipocytes, making them a valuable in vitro model for studying adipogenesis (Figure 1A). Firstly, we investigated the expression patterns of BMP8B during 3T3-L1 preadipocyte differentiation. Notably, there was a noticeable decline in the expression of BMP8B during the later stage (Figure 1B). Successful overexpression or knockdown of mouse BMP8B in 3T3-L1 cells was achieved (Figure 1C,D). Subsequently, we conducted an investigation of lipid accumulation using Oil Red O staining to assess the impact of BMP8B on adipogenesis. Overexpression of BMP8B significantly inhibited the production of lipids during adipocyte differentiation compared to the control (Figure 1E,F), along with a considerable reduction in the expression of key adipogenic marker genes (C/EBPα, PPARγ, and FASN) in both mRNA and protein levels (Figure 1G–M). In addition, we knocked down BMP8B in 3T3-L1 preadipocytes using shRNAs and subjected the cells to differentiation. On Day 8, BMP8B knockdown cells (LV-shRNA-*Bmp8b#1*), compared with control cells, showed much heavier Oil Red O staining (Figure 2A,B). Furthermore, knocking down mouse BMP8B resulted in a significant increase in C/EBPα, PPARγ, and FASN mRNA expression (Figure 2C–F). When BMP8B was knocked down, the protein levels of C/EBPα and PPARγ were also increased (Figure 2G–I). These findings suggest that BMP8B can inhibit adipogenesis.

### 3.2. BMP8B Activates SMAD2/3 Signaling to Represses Adipogenesis

**Figure 3:**
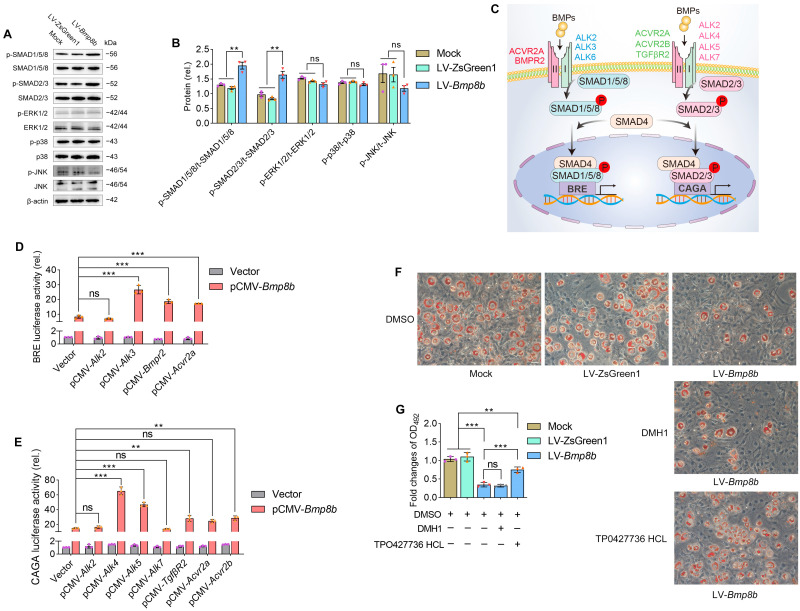
BMP8B triggers SMAD2/3 signaling to suppress adipogenesis. (**A**,**B**) Analysis using immunoblotting and quantification was conducted to assess the protein levels of p-SMAD1/5/8, p-SMAD2/3, p-ERK1/2, p-p38 MAPK, and p-JNK in LV-*Bmp8b*. (**C**) A model of BMPs-associated signal transduction. (**D**) Quantification was performed to determine the luciferase reporter activity driven by BRE, which pCMV-*Bmp8b* cotransfected with pCMV-*Alk2*, pCMV-*Alk3*, pCMV-*Bmpr2*, pCMV-*Acrv2a*, respectively. (**E**) Quantification was performed to determine the luciferase reporter activity driven by CAGA, which pCMV-*Bmp8b* cotransfected with pCMV-*Alk2*, pCMV-*Alk4*, pCMV-*Alk5*, pCMV-*Alk7*, pCMV-*Tgfβr2*, pCMV-*Acrv2a*, and pCMV-*Acrv2b*, respectively. (**F**,**G**) In the presence of DMH1 or TP0427736 HCL, the cells were induced to differentiate into adipocytes. On Day 8, Oil Red O staining was performed (**F**). Quantification of lipid content after adipogenic differentiation (**G**). Scale bar = 20 µm. The symbols in the charts represent three biological replicates. The data were presented as mean ± SD and analyzed using one-way ANOVA (ns not significant, ** *p* < 0.01, *** *p* < 0.001).

The activation of BMP signals can occur through both Smad-dependent pathways (SMAD1/5/8 and SMAD2/3) and Smad-independent pathways (such as ERK, JNK, and p38MAPK) [34,35,36]. To investigate the activation of these signals, immunoblot assays were performed on *Bmp8b*-overexpressing 3T3-L1 cells. The analysis revealed that BMP8B can activate both SMAD1/5/8 and SMAD2/3 pathways. However, there was no discernible impact on the activation status of ERK, JNK, or p38 MAPK pathways (Figure 3A,B). 

BMPs utilize various receptor complexes to activate transduction signaling pathways. For example, BMPs attach to receptor complexes developed by type I receptors (activin receptor-like kinase 2 (ALK2), ALK3, or ALK6) and type II receptors (BMPR2 or activin A receptor type 2A (ACVR2A)), which triggers SMAD1/5/8 signaling (Figure 3C) [37]. Moreover, BMPs bind to receptor complexes formed by type I receptors (ALK2, ALK4, ALK5, or ALK7) and type II receptors (TGF-β receptor 2 (TGFβR2), ACVR2A, or ACVR2B), which triggers SMAD2/3 signaling (Figure 3C) [38]. The signaling of SMAD1/5/8 activates the BRE promoter, while the signaling of SMAD2/3 activates the CAGA promoter [30,39]. To further investigate the BMP8B-mediated signal pathway, we conducted luciferase reporter assays driven by BRE and CAGA. It was found that the BRE-driven luciferase activity is significantly increased and cotransfected with BMP8B and type I receptor ALK3 or type II receptor (BMPR2 or ACVR2A) (Figure 3D). Likewise, overexpression of ALK4 and ALK5 or TGFβR2, BMPR2, and ACVR2B receptors enabled BMP8B to activate CAGA-driven luciferase activity (Figure 3E). Thus, BMP8B can trigger SMAD1/5/8 and SMAD2/3 signaling, which is consistent with the immunoblot assay results.

Then, we evaluated the impact of DMH1, which is an inhibitor for SMAD1/5/8, and TP0427736 HCl, an inhibitor for SMAD2/3, on the level of lipids in 3T3-L1 cells overexpressing BMP8B. Clearly, only the SMAD2/3 inhibitor effectively prevented the decrease in lipids in LV-*Bmp8b* cells (Figure 3F,G), indicating that BMP8B primarily inhibits adipogenesis through SMAD2/3 signaling, although BMP8B can trigger SMAD1/5/8 and SMAD2/3 signaling.

### 3.3. BMP8B Triggers SMAD2/3 Signaling to Suppress Adipocyte Differentiation via ALK4

**Figure 4:**
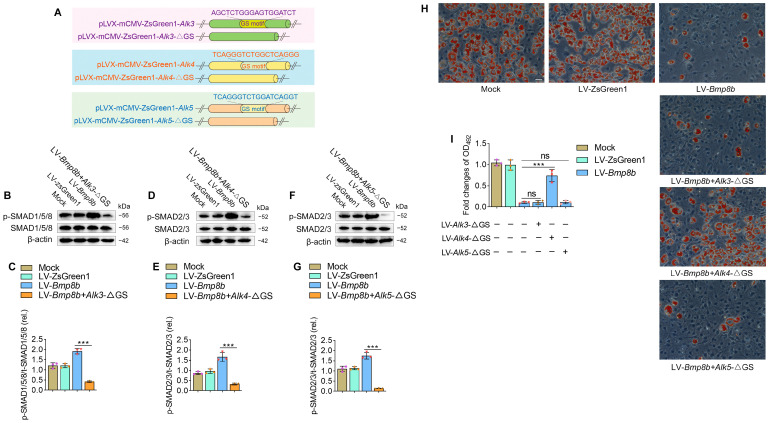
BMP8B triggers SMAD2/3 signaling to suppress adipose differentiation via ALK4. (**A**) Schematic representation of wild-type and GS motif mutants. (**B**,**C**) Western blots and quantification of p-SMAD1/5/8 in Mock, LV-ZsGreen1, LV-*Bmp8b*, and LV-*Bmp8b* +*Alk3*-ΔGS cells. (**D**,**E**) Western blots and quantification of p-SMAD2/3 in Mock, LV-ZsGreen1, LV-*Bmp8b*, and LV-*Bmp8b* + *Alk4*-ΔGS cells. (**F**,**G**) Western blots and quantification of p-SMAD2/3 in Mock, LV-ZsGreen1, LV-*Bmp8b*, and LV-*Bmp8b* + *Alk5*-ΔGS cells. (**H**,**I**) Knock down of ALK3, ALK4, and ALK5 in LV-*Bmp8b*. On Day 8, cells were stained with Oil Red O for quantification. Scale bar = 20 µm. The symbols in the charts represent three biological replicates. The data were presented as mean ± SD and analyzed using one-way ANOVA (ns not significant, *** *p* < 0.001).

Since type I receptors play a key role in BMP signaling, we investigated which type I receptors are involved in BMP8B’s inhibition of adipocyte differentiation. As mentioned above, BMP8B can activate the type I receptors ALK3, ALK4, or ALK5 (Figure 3D,E). At present, there is no commercial chemical inhibitor that can inhibit the activity of ALK3, ALK4, or ALK5, respectively. Considering that the phosphorylation of the type I receptor relies on the activation of the Gly-Ser (GS) domain [30], we designed dominant-negative mutant plasmids (*Alk3*-ΔGS, *Alk4*-ΔGS, and *Alk5*-ΔGS) (Figure 4A). Knockdown of ALK3 in LV-*Bmp8b* selectively inhibited phosphorylation of the SMAD1/5/8 (Figure 4B,C). Likewise, knockdown of ALK4 or ALK5 in LV-*Bmp8b* selectively inhibited BMP8B-mediated phosphorylation of the SMAD2/3 (Figure 4D–G). These findings are consistent with the above study (Figure 3D,E). However, only knockdown ALK4 significantly diminished the suppressive effect of BMP8B on adipocyte differentiation (Figure 4H,I), indicating that BMP8B may primarily bind to the ALK4 to trigger SMAD2/3 signaling to suppress adipogenesis.

### 3.4. BMP8B Activates SMAD2/3 Signaling to Suppress the Expression of PPARγ

**Figure 5:**
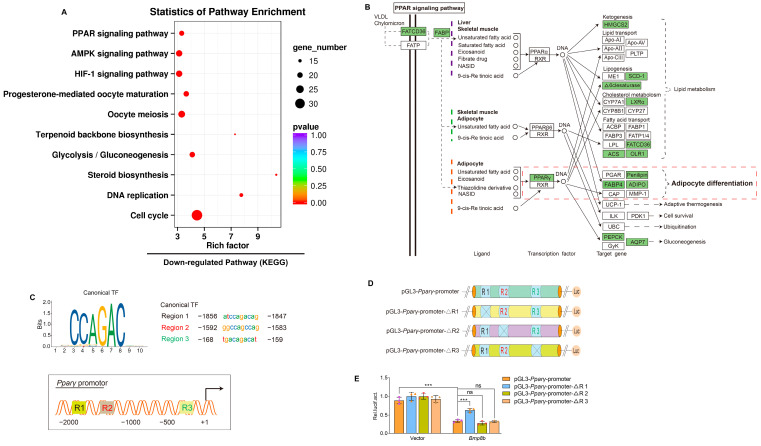
BMP8B represses PPARγ transcription via SMAD2/3 signaling, resulting in a block to adipocyte differentiation. (**A**) After induction of adipogenic differentiation, the KEGG pathway was downregulated in LV-*Bmp8b* vs LV-ZsGreen1 3T3-L1 cells. (**B**) The KEGG mapper showed downregulated genes (rectangles highlighted in green) in the PPAR signaling pathway. (**C**) The PPARγ promoter region is shown in a schematic representation. Three binding sites and sequences of SMAD2/3 TF are predicted. (**D**) Wild-type and mutation plasmids of predicted SMAD2/3 TF binding sites are shown in a schematic drawing. (**E**) Quantification the activity of pGL3-*Pparγ*-promoter and mutation promoter plasmids with pCMV-*Bmp8b* or vectors in HEK293T. *Renilla* was used as the internal control. The symbols in the charts represent three biological replicates. The data were presented as mean ± SD and analyzed using one-way ANOVA (ns not significant, *** *p* < 0.001).

In order to obtain comprehensive molecular insights into the role of BMP8B in the process of adipogenesis, we conducted transcriptome analysis on LV-*Bmp8b* and LV-ZsGreen1. By utilizing KEGG analyses, we discovered that the genes demonstrating decreased expression in LV-*Bmp8b* were significantly enriched in the PPAR signaling pathway (Figure 5A). PPAR can be divided into three types: PPARα, PPARβ, and PPARγ. Among them, PPARγ is predominantly expressed in adipose tissue and functions as the primary transcription factor responsible for regulating adipocyte formation and ensuring the mature function of adipocytes [40]. In addition, the KEGG pathway mapper illustrated that within the PPAR pathway, the expression levels of both PPARγ and its target genes (FABP4, adiponectin (ADIPO), and penilpin) exhibited a decrease (Figure 5B). The transcriptomics data closely align with the observed effects of BMP8B on the differentiation of adipocytes, at least in part, indicating that BMP8B may play a role in regulating adipogenesis by impacting the expression of PPARγ and its associated genes. BMP8A has been shown to suppress PPARγ expression by activating the SMAD2/3 pathway [30]. We next sought to identify whether BMP8B also inhibits PPARγ expression via SMAD2/3 signaling. We analyzed the PPARγ promoter region and identified three potential binding sites for SMAD2/3 (Figure 5C). We constructed pGL3-*Pparγ*-promoter and mutant plasmids (Figure 5D). Our results indicated that overexpression of BMP8B inhibits the activity of the PPARγ promoter. However, mutation of Region 1 decreased BMP8B-induced PPARγ promoter activity (Figure 5E). These data demonstrated that BMP8B activates SMAD2/3 signaling to suppress the expression of PPARγ.

### 3.5. BMP8B Triggers NF-κB Signaling to Suppress 3T3-L1 Adipocyte Differentiation

**Figure 6:**
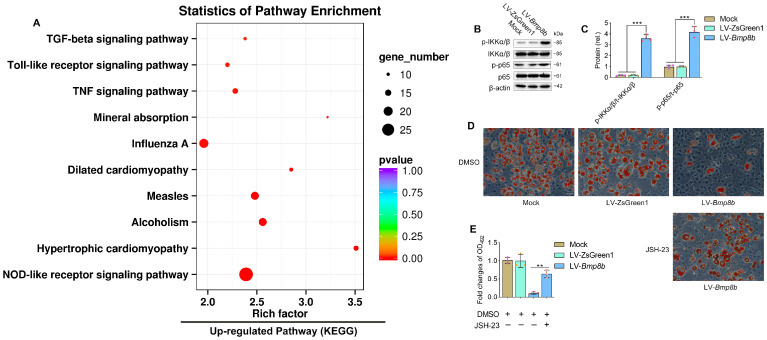
BMP8B triggers NF-κB signaling to suppress adipogenesis. (**A**) The upregulated KEGG pathway in LV-*Bmp8b* vs LV-ZsGreen1. (**B**,**C**) Western blots and quantification of p-IKKα/β and p-p65 in Mock, LV-ZsGreen1, and LV-*Bmp8b*. (**D**,**E**) Representative photographs of Oil Red O staining were taken to visualize lipids in LV-*Bmp8b* exposed to JSH-23 with DMSO as a vehicle. The staining intensity was quantified by measuring the optical density at OD_492_. Scale bar = 20 µm. The symbols in the charts represent three biological replicates. Mean ± SD was used to present the data, which were analyzed using one-way ANOVA (** *p* < 0.01, *** *p* < 0.001).

Transcriptome analysis also revealed that the up-regulated expressed genes were significantly enriched in the NOD-like receptor and TNF pathways, which play crucial roles in immunity (Figure 6A). We were curious to investigate whether BMP8B could activate NF-κB signalling, given that NF-κB signalling is downstream of both the NOD-like receptor and TNF pathways. Our results demonstrated a substantial increase in the phosphorylation levels of IKKα/β and p65 in LV-*Bmp8b* (Figure 6B,C), supporting our speculation. Furthermore, in the presence of JSH-23, an NF-κB inhibitor, the capacity of BMP8B to impede adipocyte differentiation was compromised (Figure 6D,E). Thus, BMP8B can also activate the NF-κB signal to inhibit adipocyte differentiation. Above, BMP8B was previously shown to inhibit PPARγ expression. Logically, this is in line with our previous report that NF-κB inhibits PPARγ expression [30]. An additional functional link between immune regulation and adipocyte differentiation is the regulation of NF-κB and PPARγ by BMP8B.

## 4. Discussion

The BMPs are a highly conserved group of signaling molecules that are secreted outside of cells and belong to the TGF-β superfamily. There are two *Bmp8* genes in *Mus musculus*, *Bmp8a* and *Bmp8b* [28]. BMP8A is enriched in WAT. We have demonstrated that BMP8A inhibits adipocyte differentiation [30]. BMP8B is enriched in BAT, and it plays an indispensable role in the regulation of thermogenesis in BAT [33]. However, BMP8B exhibits low expression in WAT, meaning little research focuses on its role in white adipocyte differentiation. This study demonstrated that BMP8B hampers adipocyte differentiation by activating the SMAD2/3 and NF-κB pathways, thereby enhancing the knowledge concerning BMP molecules’ functions.

The process of differentiating preadipocytes into mature adipocytes is controlled by a series of transcriptional events [41]. White adipocyte differentiation relies heavily on two crucial factors, C/EBPα and PPARγ [42]. In this study, we have demonstrated the inhibitory effects of BMP8B on adipogenesis. It is likely that BMP8B serves as a negative feedback control at the autocrine level to restrain adipogenesis because obesity leads to an increase in BMP8B [43]. Conceivably, disruption of the inhibitory regulation of BMP8B on adipogenesis is a potential contributing factor to the development of obesity. Our findings suggest that increasing the level of BMP8B or enhancing BMP8B signaling may hold potential in combatting obesity.

BMPs exert their signaling activity by adhering to BMP receptors, namely, BMPR type I (ALK2, ALK3, ALK4, ALK5, ALK6, and ALK7) and BMPR type II (BMPR2, ACVR2A, ACVR2B, and TGFBR2) [37,38]. Various functions are carried out by BMPs as they connect with distinct BMP receptors. It is commonly understood that the BMP-BMP receptors convey signals via both SMAD-dependent pathways, namely, SMAD1/5/8 and SMAD2/3 pathways, as well as SMAD-independent pathways, which include ERK, JNK, and p38 MAPK pathways [34,35,36]. Here, our findings demonstrated BMP8B activates SMAD1/5/8 signaling via ALK3 and BMP8B triggers SMAD2/3 signaling through ALK4 or ALK5. Previous research has revealed that the activation of the SMAD1/5/8 signaling pathway is crucial for promoting adipogenic differentiation in 3T3-L1 cells, while the presence of SMAD2/3 may act as an inhibitor to attenuate the adipogenesis process mediated by SMAD1/5/8 [44]. The study’s findings indicate that BMP8B activates both SMAD2/3 and SMAD1/5/8 signalling pathways. However, neither inhibition of SMAD1/5/8 through DMH1 nor knockdown of ALK3 significantly impacted the reduction in lipids in LV-*Bmp8b*; however, SMAD2/3 inhibitors and knockdown of ALK4 did. Therefore, we concluded that BMP8B activates SMAD2/3 signalling to inhibit adipogenesis via ALK4. It is imperative to conduct further investigations to comprehensively comprehend the regulatory mechanism of BMP8B in adipocyte differentiation.

Transcriptome analysis revealed that the overexpression of BMP8B significantly increased NOD-like receptor, Toll-like receptor, influenza A, and TNF signaling (Figure 6A), indicating a close association between BMP8B and immune processes. This supports previous reports that both immune cells and adipocytes are involved in antiviral responses [45]. Additionally, virus stimulation suppresses adipogenic differentiation [45]. However, the mechanism behind the inhibition of preadipocyte differentiation upon virus stimulation is still unknown. It is worth noting that NF-κB serves as the downstream signal for both the NOD-like receptor and the TNF pathway. Studies have shown that the binding of NF-κB to PPARγ prevents the activation of its target gene FABP4, consequently inhibiting the differentiation of adipocytes [30,46]. In our investigation, we have uncovered that BMP8B has the ability to activate the NF-κB signal. Therefore, we suspect that BMP8B impairs the expression of PPARγ by increasing the NF-κB signal, resulting in the inhibition of adipogenesis. This discovery could potentially establish a new connection between adipocyte differentiation and immune regulation in 3T3-L1 cells.

## 5. Conclusions

**Figure 7:**
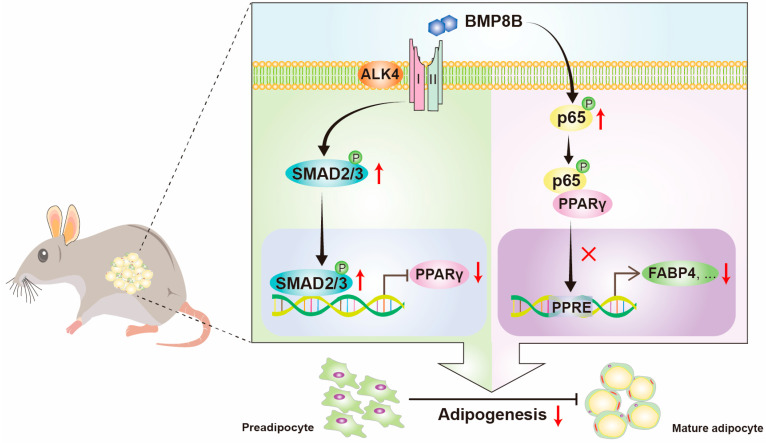
Schematic illustration of BMP8B’s role in modulating adipocyte differentiation. BMP8B binds to the ALK4 to activate SMAD2/3 signaling to suppress the expression of PPARγ to inhibit adipogenesis. Additionally, BMP8B can trigger the NF-κB signal to decrease the activation of PPARγ to inhibit adipogenesis.

Our study revealed that BMP8B inhibits adipocyte differentiation via the SMAD2/3 pathway. Additionally, overexpression of BMP8B significantly upregulates the NF-κB signal, leading to a reduction in PPARγ expression and consequent suppression of adipocyte differentiation (Figure 7). These discoveries present a fresh perspective on the function of BMP8B in mediating adipogenesis.

## Figures and Tables

**Table 1:** Oligonucleotides used in this study.

Primer	Sequences
*Gapdh*-F	GGCTGCCCAGAACATCAT
*Gapdh*-R	CGGACACATTGGGGGTAG
*Pparγ*-F	CATCCAAGACAACCTGCTGCA
*Pparγ*-R	TGACGATCTGCCTGAGGTCTGT
*C/ebpα*-F	CAAGAACAGCAACGAGTACCG
*C/ebpα*-R	GTCACTCGTCAACTCCAGCAC
*Fasn*-F	TGTGCCCGTCGTCTATACCACT
*Fasn*-R	CAATGGAAATGGCCGCTTG
*Bmp8b*-F	ATGCCAACAGTTTCCCTGATCC
*Bmp8b*-R	TTCTCCTCACCCTGCCATACC

**Table 2:** Primers used in this study.

Vector	Primer	Sequences	Applications
pCMV-C-Flag	*Alk2*-F	CGCTCTAGCCCGGGCGGATCCGCCACCATGGTCGATGGAG	Eukaryotic expression
	*Alk2*-R	TTCCTGCAGAAGCTTGGATCCACAGTCAGTTTTTAATTTGTCTAGGGAA	
	*Alk3*-F	CGCTCTAGCCCGGGCGGATCCGCCACCATGACTCAGCTATACACT	
	*Alk3*-R	TTCCTGCAGAAGCTTGGATCCAATCTTTACATCCTGGGATTCAACC	
	*Alk4*-F	CGCTCTAGCCCGGGCGGATCCGCCACCATGGCGGAGTCG	
	*Alk4*-R	TTCCTGCAGAAGCTTGGATCCAATCTTCACATCTTCCTGCACGC	
	*Alk5*-F	CGCTCTAGCCCGGGCGGATCCGCCACCATGGAGGCGGCG	
	*Alk5*-R	TTCCTGCAGAAGCTTGGATCCCATTTTGATGCCTTCCTGTTGG	
	*Alk7*-F	CGCTCTAGCCCGGGCGGATCCGCCACCATGACCCCAGCG	
	*Alk7*-R	TTCCTGCAGAAGCTTGGATCCAGCTTTACAGTCTTCCTTGACACACA	
	*Tgfβr2*-F	CGCTCTAGCCCGGGCGGATCCGCCACCATGGGTCGGGGG	
	*Tgfβr2*-R	TTCCTGCAGAAGCTTGGATCCTTTGGTAGTGTTCAGCGAGCC	
	*Acvr2a*-F	CGCTCTAGCCCGGGCGGATCCGCCACCATGGGAGCTGCT	
	*Acvr2a*-R	TTCCTGCAGAAGCTTGGATCCTAGACTAGATTCTTTGGGAGGAAAGTC	
	*Acvr2b*-F	CGCTCTAGCCCGGGCGGATCCGCCACCATGACGGCGCCC	
	*Acvr2b*-R	TTCCTGCAGAAGCTTGGATCCGATGCTGGACTCTTTAGGGAGCA	
	*Bmpr2*-F	CGCTCTAGCCCGGGCGGATCCGCCACCATGACTTCCTCGC	
	*Bmpr2*-R	TTCCTGCAGAAGCTTGGATCCCAGACAATTCATTCCTATATCTTTAGACAC	
	*Bmp8b*-F	CGCTCTAGCCCGGGCGGATCCGCCACCATGGACAGACACGA	
	*Bmp8b*-R	TTCCTGCAGAAGCTTGGATCCTAAACAGCCACAATTCTTGACCA	
pLVX-mCMV-ZsGreen1-Puro	pLVX-*Bmp8b*-F	GGTACCGCGGGCCCGGGATCCGCCACCATGGCTGCGCGT	Stable transfection
	pLVX-*Bmp8b*-R	GCAAATACGCGTCGCGGATCCGTGGCAGCCACAGGCCTG	
pLVX-shRNA2-Puro	shRNA-scrambled-F	TTGTGGAAAGGACGAGGATCCCCGGCCTAAGGTTAAGTCGC	
	shRNA-scrambled-R	ATTCGAAGCTTGTCCGGATCCCAAAAACCTAAGGTTAAGTCGCCC	
	shRNA-*Bmp8b#1*-F	TTGTGGAAAGGACGAGGATCCGCTCTACTATGATAGAAACAATTCAAGAGA	
	shRNA-*Bmp8b#1*-R	ATTCGAAGCTTGTCCGGATCCAAAAAAGCTCTACTATGATAGAAACAATCTC	
	shRNA-*Bmp8b#2*-F	TTGTGGAAAGGACGAGGATCCGCCTTTCATGGTTGGTTTCTTT	
	shRNA-*Bmp8b#2*-R	ATTCGAAGCTTGTCCGGATCCAAAAAAGCCTTTCATGGTTGGT	
	shRNA-*Bmp8b#3*-F	TTGTGGAAAGGACGAGGATCCGCTGACCTGATTATGAGCTTTTTCA	
	shRNA-*Bmp8b#3*-R	ATTCGAAGCTTGTCCGGATCCAAAAAAGCTGACCTGATTATGAGCT	
pLVX-mCMV-ZsGreen1-Puro	pLVX-*Alk3*-F	GGTACCGCGGGCCCGGGATCCGCCACCATGACTCAGCTATACACT	Dominant negative mutant
	pLVX-*Alk3*-R	GCAAATACGCGTCGCGGATCCTCAAATCTTTACATCCTGGGATTCA	
	pLVX-*Alk4*-F	GGTACCGCGGGCCCGGGATCCGCCACCATGGCGGAGTCG	
	pLVX-*Alk4*-R	GCAAATACGCGTCGCGGATCCTTAAATCTTCACATCTTCCTGCACG	
	pLVX-*Alk5*-F	GGTACCGCGGGCCCGGGATCCGCCACCATGGAGGCGGCG	
	pLVX-*Alk5*-R	GCAAATACGCGTCGCGGATCCTTACATTTTGATGCCTTCCTGTTG	
	pLVX-*Alk3*-△GS-F	CCAGTCCCAATTGCCTTTATTGGTTCAGCGAAC	
	pLVX-*Alk3*-△GS-R	AAAGGCAATTGGGACTGGTCAATCAGGTCTTTC	
	pLVX-*Alk4*-△GS-F	TCTCCACGTTACCCCTTTTTGTCCAGCGCACAG	
	pLVX-*Alk4*-△GS-R	AAAGGGGTAACGTGGAGAGGTCGTAGACGAGAT	
	pLVX-*Alk5*-△GS-F	TGACAACATTACCACTGCTTGTTCAAAGAACAA	
	pLVX-*Alk5*-△GS-R	GCAGTGGTAATGTTGTCATATCATAAATTAAATCTTTTAAGG	
pGL3-basic	pGL3-*Pparγ*-F	TGGTAAAATCGATAAGGATCCAACAAACAGACAAAGGAAGGAAATAA	Luciferase assay
	pGL3-*Pparγ*-R	AGGGCATCGGTCGACGGATCCGGAGGCCCCGCGCCCGCA	
	pGL3-*Pparγ*-△R1-F	GGGTAGAAAAGTCTAAAGTACATGGATGGTGAACCAAG	
	pGL3-*Pparγ*-△R1-R	CTTTAGACTTTTCTACCCTAGATATTTTCTATAAATG	
	pGL3-*Pparγ*-△R2-F	AGACGATATAGCAAGACCTTTTCAAAAAGTTTA	
	pGL3-*Pparγ*-△R2-R	GGTCTTGCTATATCGTCTTGAACTTATTGTTATTCTCCTAAGGCC	
	pGL3-*Pparγ*-△R3-F	ACTTCTCCAGGACATGGACATCGGTCTGAGGGA	
	pGL3-*Pparγ*-△R3-R	TCCATGTCCTGGAGAAGTTTGTTTTTTTCCTAGATG	

## Data Availability

Upon reasonable request, all data will be made available by the corresponding author. The data are not publicly available due to privacy.
